# Predictive Model of Dynamic Subphenotypes for 30-Day Mortality in Emergency Department Patients with Suspected Infection Using the Vital Signs of the First 24 Hours: An Analytical Cohort Study in a Tertiary Care Clinic

**DOI:** 10.3390/jcm15062264

**Published:** 2026-03-17

**Authors:** Alvaro Patiño-Moncayo, Milcíades Ibañez-Pinilla, Manuel Gonzalez-Varela, Juan Carlos Martinez, Luisa Fernanda Patiño-Unibio

**Affiliations:** 1 Especialización en Medicina Interna, Facultad de Medicina, Fundación Universitaria Sanitas, Bogotá 110111, Colombia; adpatinom@unal.edu.co (A.P.-M.); mfgonzalezva@unisanitas.edu.co (M.G.-V.); jcmartinezac@unisanitas.edu.co (J.C.M.); 2 Grupo de Investigación Traslacional, Unidad de Investigación, Facultad de Medicina, Fundación Universitaria Sanitas, Bogotá 110111, Colombia; mibanezpi@unisanitas.edu.co; 3 Grupo de Investigación de Medicina Crítica y Cuidado Intensivo, Department of Emergency Medicine, Clínica Colsanitas S.A., Clínica Universitaria Colombia, Bogotá 110111, Colombia; 4 Grupo de Investigación de Medicina Crítica y Cuidado Intensivo, Department of Internal Medicine, Clínica Colsanitas S.A., Clínica Universitaria Colombia, Bogotá 110111, Colombia; 5 Grupo de Investigación INPAC, Department of Internal Medicine, Clínica Colsanitas S.A., Clínica Universitaria Colombia, Bogotá 110111, Colombia

**Keywords:** sepsis, critical care, survival, mortality, signs and symptoms, phenotype, septic shock

## Abstract

**Background/Objectives**: To determine and validate the cluster trajectory model by dynamic subphenotypes of vital signs in infecti ons and prediction of 30-day mortality. **Methods:** We conducted a prospective study in the emergency department of Clínica Colombia with patients with suspected infection. Clinical data, vital signs, and 30-day mortality were collected. Vital signs were measured within the first 24 h of admission, and patients were classified according to a vital signs trajectory model into four groups: A, B, C, and D. **Results**: The final cohort consisted of 625 patients, and the subphenotypes, according to the vital signs, were as follows: A—2.66% (all vital signs altered), B—9.2% (at least one alteration, predominantly hypertension), C—19% (minimal or no alteration, control), D—69.2% (only arterial hypotension). The primary outcome was mortality. Overall mortality was 8.6%, being higher in group A followed by D, B and C (*p* = 0.009). The risk increased progressively in groups B (OR = 2.87, CI 95%: 0.62–13.25), D (OR = 4.77, CI 95%: 1.46–15.58), and A (OR = 8.33, CI 95%: 1.54–45.05). Group A presented more frequently with pneumonia (*p* = 0.002), CNS infections (*p* = 0.021), mechanical ventilation (*p* < 0.001), and vasopressor support (*p* < 0.001). Significant differences among groups were found in leukocytosis (B vs. C *p* = 0.026), neutrophilia (B vs. C *p* = 0.001 and B vs. D *p* = 0.042), lymphocytosis (B vs. D *p* = 0.002), neutrophil/lymphocyte ratio (A vs. C *p* = 0. 010), lactate (C vs. D *p* = 0.044), anemia (B vs. D *p* = 0.013 and C vs. D *p* = 0.001), and CRP (A vs. C *p* = 0.004, B vs. C *p* < 0.001 and C vs. D *p* < 0.001). **Conclusions**: Patients with suspected infection and more altered vital signs have higher mortality (group A) and benefit from earlier interventions by sepsis teams in the emergency department.

## 1. Introduction

Sepsis is a heterogeneous syndrome characterized by a dysregulated host response to infection. In the United States, it causes 700,000 hospitalizations, 270,000 deaths, and $60 billion in total costs each year [1]. The best way to optimize the prognosis for these patients is to recognize them at the earliest stage [2]. Most of the sepsis subphenotype models that have been developed have used data from patients admitted to the emergency department with high SOFA scores and established organ dysfunction. Likewise, biomarkers such as IL6, CRP, and lactate are the most common in clinical practice. Markers can clarify the diagnosis and prognosis in patients with a high mortality rate; however, biomarkers contribute to the diagnosis and prognosis of patients. The increase in biomarkers can be a late manifestation and absent during the first encounter with the patient in the emergency department [2]. Bhavani et al. conducted a study using vital sign trajectories to develop phenotypes for 12,473 patients with suspected infection, finding that this model can be used in patients with and without established sepsis as a dynamic process with different mortality rates depending on each phenotype allows us to characterize phenotypes based on vital signs in patients with sepsis [3]. This provides insight into the dynamic process and patient behavior in a local setting close to our reality.

The implementation of sepsis rapid response teams relies on tools that enable rapid decision-making to impact patient outcomes. The vital signs to establish trajectories that help predict the course of the disease in real time [4].

The growing development of artificial intelligence in medical record software is promising. Stratifying the risk of patients with infection or suspected infection with a high probability of progression to sepsis and death using vital sign trajectories or biomarkers such as lactate and alarm systems in electronic medical records could more accurately and quickly predict the groups of patients in which a sepsis team could intervene in order to improve clinical outcomes and hospital costs [5].

Therefore, it is necessary to understand dynamic phenotypes based on vital sign measurement, a tool easily obtained in emergency departments, which undoubtedly changes the clinical approach and patient care times. This study proposes applying a group-based trajectory model (GBTM) to a prospective cohort of patients admitted to the emergency department with suspected infection of any origin to identify phenotypes that exhibit similar progression within the first 24 h of admission to the emergency department and to identify the association with mortality and advanced life support [6].

The management of patients with infection in the emergency department is challenging, which has led to the description of patient subphenotypes to achieve a better understanding of the interindividual and interpopulation behavior of individuals with infection and its association with outcomes such as mortality [7].

Despite the importance of this topic, the existing literature and research appear to be very limited in terms of prospective studies that validate which dynamic phenotypes of sepsis are associated with higher mortality in our population. Therefore, finding an association would have a major impact on the risk management of patients with sepsis, their morbidity and mortality, hospital costs, and high-quality standards for a fourth-level care institution.

## 2. Materials and Methods

An observational, analytical, prospective cohort study was conducted with patients over 18 years of age with suspected infection in the emergency department of the Clínica Universitaria Colombia, excluding those under 18 years of age, pregnant women, those who had died within the first 24 h, or those receiving end-of-life care. For the purposes of the study, suspected infection was defined as the presence of signs or symptoms indicating an active infectious process, as determined by the first-contact physician. Patients were recruited between January and December 2024. The estimated sample size was 675 patients. The estimated sample size, with a statistical power of 80% and a reliability of 95%, was 675 patients for an association between mortality and vital sign phenotypes in patients with suspected infection as the primary endpoint and need for inotropic or vasopressor support, renal replacement therapy, and mechanical ventilation within the first 72 h of patient admission as the secondary endpoints. Vital signs were measured and monitored in the first 24 h of admission (heart rate, respiratory rate, blood pressure, and temperature).

The group-based trajectory model (GBTM) is a finite mixture model used to identify groups of patients or individuals who have a similar trajectory of a variable over a set period. The algorithm calculates the underlying coefficients of polynomial functions describing certain variables, which in our study were the trajectories of vital signs in the first 24 h of ED admission for each of the groups. Before adjusting the model, a likelihood ratio test was used to determine the polynomial form that best fit each of the vital signs (linear versus quadratic). Using GBTM, the polynomial forms selected for each of the vital signs were prespecified, and one, two, three, and four-group models were tested. The resulting models with varying numbers of subgroups or subphenotypes were compared using Bayesian information criterion (BIC), a metric of how well the model fits the data, with a penalty for increasing complexity, and distribution by subphenotype. If one or more subphenotypes contained less than 5% of the cohort, the model was not amenable to selection for analysis. Once the optimal model was selected, patients were assigned the subphenotype for which they had a higher probability of belonging. Model fit was assessed by ensuring that the mean probability of group membership was >70% for each of the subphenotypes. Subphenotypes were defined by a set of five unique polynomial functions that described each vital sign as a function of time elapsed from the time of ED admission (i.e., Temperature = β0 + β1 × Time + β2 × Time2) [1].

The information was entered using a data collection tool into an Excel version 2506 database for Microsoft 365, which included the dependent variable (mortality), the independent variables (vital signs taken every 4 h in the first 24 h after admission to the emergency department), from which the subphenotypes of vital sign trajectories were constructed; and confounding variables such as demographic variables, patient characteristics, support requirements, comorbidities, type of infection, and laboratory tests (see Table S1). Information was collected through interviews with patients and/or caregivers after signing an informed consent form and by reviewing medical records in the electronic system of the Clínica Universitaria Colombia (Sophia and Avicena).

An audit of the information stored in the data collection instrument was also carried out by reviewing medical records three times a week to verify the accuracy and consistency of the data, without imputing missing data because the group-based trajectory model handles missing data through probability estimation; in the case of multiple measurements at the same time, the average measurement was used for subsequent analysis.

The information was then refined and processed using SPSS version 25.0 and Stata version 15.0. Absolute and relative frequency measures were calculated as percentages, and for quantitative variables, measures of central tendency such as mean and median, measures of dispersion such as range and standard deviation, and measures of dispersion such as percentiles and quartiles were calculated. Normality was assessed using the Shapiro–Wilk and Kolmogorov–Smirnov tests.

The incidence of vasopressors, inotropic, ventilatory, and renal support requirements was measured as a percentage. The trajectory groups were constructed into independent categories and measured with absolute and percentage frequency distributions. Bivariate analysis was performed for the categorical variable of the trajectory groups (the other clinical and demographic variables) with 30-day mortality using Pearson’s chi-square test or asymptotic or exact likelihood ratio (expected values z < 5), adjusting the model to ensure that the average posterior probability of belonging to a subphenotype was greater than 70%.

Differences in demographic characteristics, comorbidities, and clinical characteristics were compared using analysis of variance (ANOVA) or chi-square tests as appropriate. In the case of laboratory data with multiple measurements on the same day, the highest values in the first 24 h were used without amputating missing data and comparing laboratory results between subphenotypes using ANOVA.

Given the set of laboratory tests included, all significant tests were corrected for multiple testing using the Bonferroni correction. Finally, an unconditional logistic regression and a negative binomial log model. The ROC curve was used to predict 30-day mortality with the subphenotypes. The significance level was set at 5% (*p* < 0.05) [1].

## 3. Results

### 3.1. General Cohort

**Figure 1:**
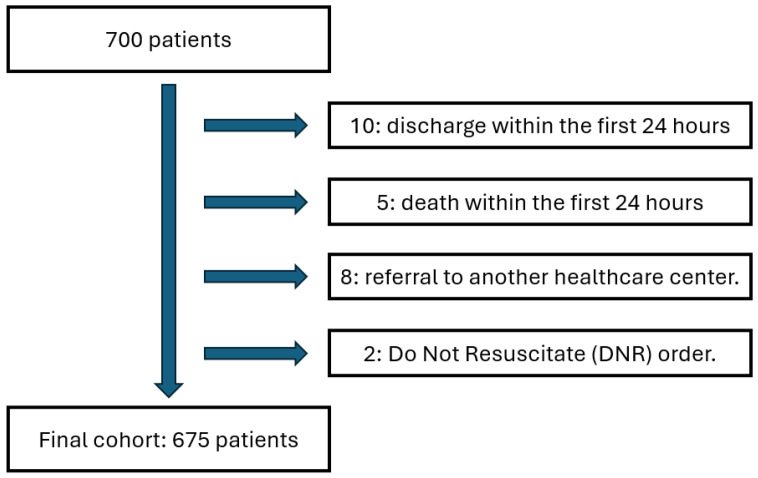
Flowchart of the selection process and final composition of the sample of patients with suspected infection in the emergency department of the Clínica Universitaria Colombia (January–December 2024).

The initial cohort consisted of 700 patients over the age of 18 who were admitted to the emergency department of the Clínica Universitaria Colombia between January and December 2024. All of them had their vital signs assessed during the first 24 h of care. Of the total, 25 patients were excluded, of whom 10 were discharged within the first 24 h, 5 died during that same period, 8 were referred to other healthcare centers, and 2 had a do-not-resuscitate order. After applying these exclusion criteria, the final sample consisted of 675 patients. In all cases, informed consent was obtained for the use of clinical information for research purposes. The identification and follow-up of each patient included in the study were carried out through the institutional electronic medical record system (Figure 1).

### 3.2. Classification by Subphenotypes

The participants in the study were divided into four groups according to the behavior of their vital signs in the first 24 h after admission to the emergency department, corresponding to the subphenotypes we call A, B, C, and D. In each group, the distribution of patients was as follows: group A, 18 patients (2.66%), corresponding to those patients who had altered vital signs (hypotension, tachycardia, tachypnea, fever); group B, 62 patients (9.2%) with at least one altered vital sign during the first 24 h, predominantly hypertension; group C, 128 patients (19%) with no or minimal alterations in vital signs (control group); and group D, 467 patients (69.2%) with only arterial hypotension.

### 3.3. Sociodemographic and Clinical Characteristics by Subphenotypes

The minimum age was 18 and the maximum age was 96, with an average of 59.2 ± 19.3 years (median = 63.0 years). Individuals were classified into five age groups: under 40, 40–49, 50–59, 60–69, and 70 or older. Group C was made up of younger individuals, and group D was made up mostly of individuals who were 70 years of age or older. Regarding sex, the analysis shows a *p*-value of 0.008, indicating a statistically significant difference between the cohorts. Group A has a majority of females (61.1%), as does group D (51.4%). In contrast, group B was predominantly composed of males (69.4%), followed by group C with 56.3% males (Table 1).

In the association between clinical variables and subphenotypes, significant differences were found in the history of hospitalization in the last 30 days, with a higher frequency in groups D and B, followed by groups C and A; a history of chronic obstructive pulmonary disease (COPD) and peripheral arterial disease was more frequent in group D; a history of connective tissue disease was much more frequent in group A; leukemia was more frequent in group B, with no cases recorded in groups A or C; in terms of frailty, it was found that individuals in group A were more frequently frail.

Although no statistically significant difference was found, groups A and D had lower physical resistance, a higher frequency of chronic kidney disease, diabetes mellitus, and higher overall comorbidity. Group A had higher frequency of fatigue, antibiotic use in the last 30 days, and heart failure. Group D required renal replacement therapy more frequently and had a higher frequency of liver disease and acute myocardial infarction; Group B had a higher frequency of cerebrovascular disease.

Regarding the type of infection, a statistically significant difference was found in pneumonia, which was more frequent in group A, while skin and soft tissue infection was more frequent in group C; central nervous system (CNS) infection was more frequent in group A. Although no statistically significant difference was found, urinary tract infection was more frequent in group B, and endocarditis and catheter-associated infection were more frequent in group A.

Regarding the need for renal replacement therapy, no statistically significant differences were found between the subphenotypes, although it was observed more frequently in group D. A statistically significant difference was found in the need for mechanical ventilation, which was more frequent in group A, with no cases documented in groups B and C. Regarding the need for inotropic/vasopressor support, a statistically significant difference was also found between group A with no cases documented in group C. These findings correlate with greater severity of infection and higher mortality in group A (Table 2).

### 3.4. Laboratory Characteristics by Subphenotypes

Statistically significant differences were found in the analysis of subphenotypes in leukocytosis, absolute neutrophil count, lymphocytosis, neutrophil/lymphocyte ratio, CRP levels, lactic acid levels, hemoglobin levels, PTT (thromboplastin time), and PAFI (ratio between oxygen pressure and inspired oxygen fraction by arterial gases) levels. Thus, groups B and D were found to have the greatest alteration in laboratory values. Although no statistically significant difference was found, higher ESR levels were observed in groups D and A. Platelet levels were lower in groups B and D; protrombin time (PT) was longer in groups D and B, and renal function, assessed by creatinine levels, was higher in patients in groups B and A. Finally, higher bilirubin levels were observed in groups D and A (Table 3).

### 3.5. Mortality Prediction by Subphenotypes

The final cohort showed an overall mortality rate of 8.6% (58 patients), demonstrating a statistically significant difference, as the group with the highest mortality rate was group A (OR 8.33, 95% CI: 1.54–45.05), followed by groups D (OR 4.77, 95% CI: 1.46–15.58), B (OR 2.87, 95% CI: 0.62–13.25), and C, respectively. In the group with the greatest impact on vital signs (group A), mortality was higher than in the other groups, with a statistically significant difference in the latter (*p* = 0.009). In other words, belonging to group A carries an 8-fold higher risk of mortality compared to groups B (2.8-fold higher risk) and D (4.7-fold higher risk) (Table 4).

**Figure 2:**
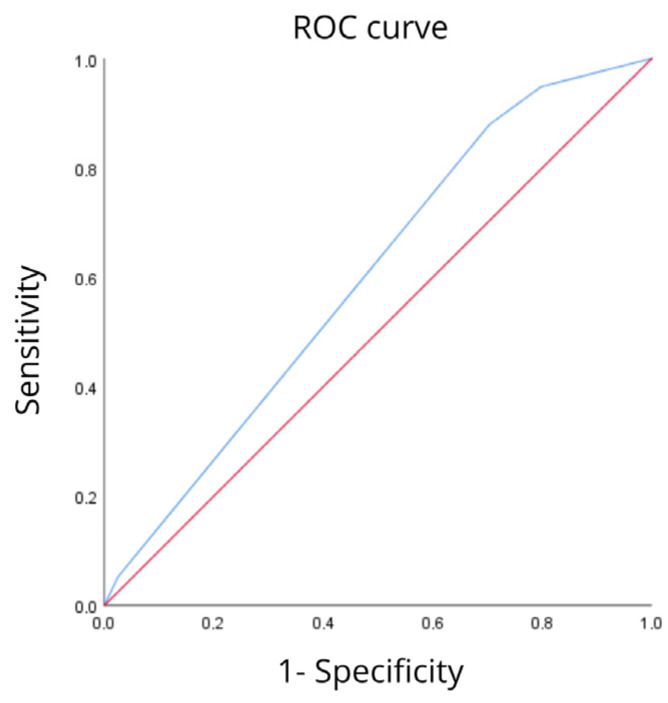
ROC curve. Subphenotypes and mortality.

The area under the curve for predicting 30-day mortality based on vital signs measured within the first 24 h of admission of patients with suspected infection to the emergency department using classification with subphenotypes with the ROC curve was 60% (95% CI: 53.1%, 66.9%, *p* = 0.012). The model’s accuracy was 60% in the subphenotypes compared to mortality, as shown in the ROC curve (see Figure 2).

## 4. Discussion

### 4.1. Characterization of Subphenotypes

To characterize clinical subphenotypes based on the behavior of vital signs in the first 24 h after admission to the emergency department, models based on clinical and laboratory data have been described, allowing for a more accurate characterization of this population based on dynamic changes that, unlike static or single measurements, enable earlier detection of information relevant to real-time decision-making [2,8].

In our study, four subphenotypes were classified, each comprising patients between the ages of 18 and 96 (A, B, C, and D), which were characterized by different vital sign behavior in the first 24 h after admission. An overall mortality rate of 8.6% was identified in the cohort, which was highest in group A, followed by groups D, B, and C (in our case, the control group). The groups were characterized by alterations in all vital sign parameters (group A), alterations in at least one vital sign along with high blood pressure (group B), no alterations (group C or control), and hypotension only (group D), with a statistically significant difference between groups A and C and D and C.

### 4.2. Association Between Subphenotypes and Mortality

It was found that mortality increases in relation to a higher burden of comorbidities and early alterations in vital signs, without laboratory alterations being a clear determinant of mortality.

In a prospective cohort study conducted by Scicluna et al. in 2017, which described endotypes of patients admitted to hospital with a diagnosis of sepsis secondary to community-acquired pneumonia in the Netherlands between June 2011 and July 2012, four endotypes (MARS 1, 2, 3, and 4) were identified based on genomic sequencing, with endotype 1 being the most associated with mortality, whose characteristics varied from those of the other patients included in the other phenotypes. Similarly, in our study, group A (which is equivalent to group 1 in the study) had the highest mortality rate, which could be related to the pathophysiological and genomic phenomena that lead to greater alteration of vital signs [9].

When comparing the study by Aldewereld et al., in which a retrospective analysis was performed on a cohort of 1023 patients taken from the ProCESS trial, where they found five phenotypes based on clinical features and 23 clinical variables (two high-risk, one moderate risk, and two low risk phenotypes) and in which the highest mortality was observed in those with high-risk phenotypes (42.4%, characterized by higher levels of multiple organ failure, SOFA and APACHE II scores, respiratory and cardiac failure) and lower mortality in the moderate-risk group (34.3%, characterized by respiratory failure, but with mild hypotension and lower vasopressor requirements) and low risk (14.6%, characterized by better response to intravenous fluids and lower incidence of multiple organ dysfunction). In our study, patients with higher mortality rates, in addition to having greater alterations in vital signs, were associated with a higher burden of comorbidities, especially in groups A and D compared to groups B and C; Similarly, patients with greater requirements for vasopressor/inotropic support and mechanical ventilation (belonging to group A) had the highest mortality rate, which is consistent with the findings of the study by Aldewerld et al. [10].

In the study by Seymour et al., four groups (phenotypes) were classified: alpha (the most common and characterized by a lower requirement for vasopressor support), beta (encompassing older patients with more comorbidities and more renal diffusion), gamma (patients with a higher degree of inflammation and pulmonary diffusion), and delta (patients with greater hepatic diffusion and septic shock). Thus, we see that groups A/delta in the Seymour study) and D showed the greatest alterations in laboratory tests and, at the same time, were associated with higher mortality, like the findings of Seymour et al. [11].

Dr. Bhavani et al. described four phenotypes based on vital signs in a manner similar to our study. They found that, when grouping and stratifying patients according to these parameters, patients in group A had hyperthermia, tachycardia, tachypnea, and hypotension; patients in group B had hyperthermia, tachycardia, tachypnea to a lesser degree than group A, and hypertension; patients in group C had hypothermia, normal heart and respiratory rates, and normotension; and finally, patients in group D had hypotension only. Groups A and B were mostly confirmed by young patients compared to groups C and D; groups A and D required greater vasopressor support. Thirty-day mortality was higher in groups A and D than in groups B and C (*p* < 0.001 and *p* < 0.003, respectively). Our results are consistent with Dr. Bhavani’s study, considering that the subphenotypes found in the cohort had higher mortality in groups A and D compared to groups B and C (in our case, the control group), with a statistically significant difference [1].

The findings show that the early evolution of vital signs is not a random phenomenon but rather reflects underlying pathophysiological patterns with relevant prognostic value. Patients classified in group A (those with combined alterations in temperature, heart rate, respiratory rate, and blood pressure) had significantly higher mortality, with an 8-fold higher risk compared to those with stable parameters. This observation highlights the importance of early identification of severity phenotypes based on simple clinical data available upon admission, such as vital signs, medical history, comorbidities, and laboratory tests [12].

In addition, structured and protocolized responses to sepsis have been associated with improved bundle adherence and better process –of care metrics. Delawder and Hulton demonstrated that the implementations of an interdisciplinary Code Sepsis team significantly improved compliance with sepsis management bundles in emergency settings [13]. In this context, phenotypic classification could enhance risk stratification and support timelier, individualized clinical decision including triage prioritization and closer monitoring in order to facilitate the early initiation of advanced therapies and team-based interventions

The use of a robust prospective cohort, with systematic collection of clinical and paraclinical data, in the context of an institution with a system capable of extensive follow-up, lends methodological strength to the results [14].

### 4.3. Strengths and Limitations

Although this is a rigorously constructed prospective cohort, the exclusion of patients who were discharged, referred, or died during the first 24 h of admission could introduce selection bias, given that these cases represent extremes of the clinical spectrum (either due to rapid resolution or early critical deterioration) that could alter the composition and behavior of the analyzed subphenotypes.

Subphenotypes were classified based exclusively on vital sign trajectories during the first 24 h, which excludes other relevant aspects in the diagnosis of infections in patients who visit the emergency department, such as serum and immunological biomarkers. However, in our study, variables related to these parameters were included, such as laboratory data and comorbidities, which, although not analyzed in direct relation to the construction of phenotypes, were observed to be related to these groups.

## 5. Conclusions

This prospective observational study allowed us to characterize, based on vital sign trajectories during the first 24 h of admission, different clinical subphenotypes in patients with suspected infection, which showed significant differences in terms of vital signs, paraclinical findings, medical history (comorbidities), and 30-day mortality, with this outcome being more frequent in group A, i.e., those patients with alterations in all vital signs, who would benefit from early interventions by sepsis and infection care teams at all levels of care, taking into account that it is based on vital signs, characteristics that are easily obtained by emergency departments and have an impact on mortality.

The findings show that early changes in vital signs reflect underlying pathophysiological patterns with significant prognostic value [12].

From an epidemiological perspective, this phenotypic approach allows for more accurate stratification of the mortality risk of patients admitted with infection or suspected infection and could guide more timely and personalized clinical decisions, such as triage prioritization, intensified monitoring, or early initiation of advanced therapies. Furthermore, the integration of these phenotypes contributes to a broader understanding of the heterogeneity in the presentation and evolution of sepsis in the emergency setting, which lays the foundation for the standardization and protocolization of sepsis response teams, as occurs in other critical conditions such as acute myocardial infarction or stroke [13].

This study not only provides evidence of the association between early vital sign patterns and mortality in patients with infection or sepsis but also proposes a practical, scalable, and clinically intuitive way to address the complexity of this condition. Identifying subphenotypes is not just an academic tool; rather, it is a way to humanize medicine through clinical precision, allowing for the timely recognition of patients who need it most and, with that, offering better opportunities for survival and recovery (68). The use of a robust prospective cohort, with systematic collection of clinical and paraclinical data, in the context of an institution with a system with a high capacity for follow-up, gives methodological strength to the results [15].

## Figures and Tables

**Table 1:** Classification by vital sign subtypes.

Subphenotypes	Gender (*n*, %)		Age (Years)					Mortality (*n*, %)	OR ^1^ for Mortality (CI 95%)
	Male	Female	<40	40–49	50–59	60–69	>70		
**A**	7 (38.9%)	11 (61.1%)	4 (22.2%)	3 (16.7%)	2 (11.1%)	6 (33.3%)	3 (16.7%)	3 (16.7%)	8.33 (1.54–45.02)
**B**	43 (69.4%)	19 (30.6%)	12 (19.4%)	9 (14.5%)	8 (12.9%)	14 (22.6%)	19 (30.6%)	4 (6.5%)	2.87 (0.62–13.25)
**C**	72 (56.3%)	56 (43.8%)	35 (27.3%)	21 (16.4%)	16 (12.5%)	26 (20.3%)	30 (23.4%)	3 (2.3%)	1
**D**	227 (48.6%)	240 (51.4%)	87 (18.6%)	41 (8.8%)	55 (11.8%)	105 (22.5%)	179 (38.3%)	48 (10.3%)	4.77 (1.46–15.58)

^1^ Odds ratio.

**Table 2:** Requirement for renal replacement therapy, mechanical ventilation, vasopressors, and inotropic agents by Subphenotypes.

Intervention	Category	A	B	C	D	Total
Renal replacement therapy within the first 48 h (*p* = 0.172)	No—Patients	18	60	122	432	632
	% within the subphenotype	100.0%	96.8%	95.3%	92.5%	93.6%
	Yes—Patients	0	2	6	35	43
	% within the subphenotype	0.0%	3.2%	4.7%	7.5%	6.4%
Mechanical ventilation in the first 48 h (*p* < 0.001)	No—Patients	15	62	128	452	657
	% within the subphenotype	83.3%	100.0%	100.0%	96.8%	97.3%
	Yes—Patients	3	0	0	15	18
	% within the subphenotype	16.7%	0.0%	0.0%	3.2%	2.7%
Vasopressor/inotropic agent in the first 24 h (*p* < 0.001)	No—Patients	15	61	128	435	639
	% within the subphenotype	83.3%	98.4%	100.0%	93.1%	94.7%
	Yes—Patients	3	1	0	32	36
	% within the subphenotype	16.7%	1.6%	0.0%	6.9%	5.3%
Total (*n*)		18	62	128	467	675

**Table 3:** Laboratory characteristics by subphenotypes.

		N	Media	Deviation	Deviation Error	95% Confidence Interval for the Mean		Minimum	Maximum
						Lower Limit	Upper Limit		
**White blood cells (*p* = 0.040)**	A	18	13,397.22	7.176.347	1.691.481	9828.51	16,965.94	2210	25,160
	B	62	15,842.74	20.797.560	2.641.293	10,561.15	21,124.33	580	149,820
	C	128	10,597.95	4.942.481	436.858	9733.49	11,462.42	2470	32,810
	D	466	12,313.32	11.696.897	541.848	11,248.55	13,378.10	100	208,000
	Total	674	12,341.17	11.890.560	458.007	11,441.87	13,240.46	100	208,000
**Absolute neutrophil count (*p* = 0.001)**	A	18	11,127.22	6.513.525	1.535.253	7888.12	14,366.32	1290	23,230
	B	62	11,994.35	12.349.497	1.568.388	8858.17	15,130.54	280	73,560
	C	128	7779.18	4.517.477	399.292	6989.05	8569.31	150	26,300
	D	466	9382.24	6.809.993	315.467	8762.33	10,002.16	0	48,800
	Total	674	9364.69	7.210.230	277.728	8819.37	9910.01	0	73,560
**Lymphocytes (*p* = 0.002)**	A	18	1017.78	770.451	181.597	634.64	1400.91	150	2500
	B	62	2329.52	5.263.661	668.486	992.80	3666.24	60	40,550
	C	128	1662.42	927.882	82.014	1500.13	1824.71	100	4770
	D	466	1400.13	1.133.023	52.486	1296.99	1503.27	0	12,360
	Total	674	1525.22	1.912.223	73.656	1380.60	1669.85	0	40,550
**Neutrophil/Lymphocyte ratio (*p* = 0.004)**	A	18	20.304.233.245.050.000	21.945.343.744.543.900	5.172.567.125.745.590	9.391.070.545.763.740	31.217.395.944.336.400	1.454.545.454.545.450	87.869.565.217.391.300
	B	62	10.001.785.697.985.300	8.321.767.798.750.330	1.056.865.567.307.380	7.888.452.383.428.810	12.115.119.012.541.800	0.150431565967941	46.000.000.000.000.000
	C	128	7.648.725.100.072.030	12.486.910.993.720.700	1.103.697.429.966.600	5.464.707.065.253.280	9.832.743.134.890.780	0.084745762711864	100.000.000.000.000.000
	D	467	11.794.591.453.901.500	17.191.887.565.804.400	0.795545680333538	10.231.290.319.135.000	13.357.892.588.668.000	0.000000000000000	174.000.000.000.000.000
	Total	675	11.070.663.605.136.600	16.034.747.135.522.600	0.617177705005440	9.858.841.417.119.850	12.282.485.793.153.400	0.000000000000000	174.000.000.000.000.000
**C-reactive protein (CRP) (*p* < 0.001)**	A	14	1.743.643	13.472.108	3.600.572	965.786	2.521.499	29.45	426.74
	B	52	1.479.892	10.247.576	1.421.083	1.194.598	1.765.187	0.49	479.00
	C	107	727.393	7.598.272	734.553	581.760	873.025	0.60	328.96
	D	366	1.396.836	10.960.943	572.938	1.284.168	1.509.503	0.00	445.10
	Total	539	1.280.962	10.731.830	462.253	1.190.157	1.371.766	0.00	479.00
**Erythrocyte sedimentation rate (ESR) (*p* = 0.703)**	A	8	38.88	32.224	11.393	11.93	65.82	5	102
	B	39	35.49	29.218	4.679	26.02	44.96	2	100
	C	80	35.25	30.069	3.362	28.56	41.94	2	120
	D	285	39.14	29.360	1.739	35.72	42.57	1	120
	Total	412	38.04	29.480	1.452	35.18	40.89	1	120
**Lactic acid (*p* = 0.030)**	A	15	1.967	17.303	0.4468	1.008	2.925	0.4	6.7
	B	45	1.422	10.473	0.1561	1.108	1.737	0.5	7.0
	C	74	1.199	0.5992	0.0697	1.060	1.337	0.3	4.0
	D	332	1.685	15.475	0.0849	1.518	1.852	0.4	17.0
	Total	466	1.591	14.118	0.0654	1.463	1.720	0.3	17.0
**Hemoglobin (*p* < 0.001)**	A	18	125.889	217.144	0.51181	115.091	136.687	7.90	17.00
	B	62	132.984	301.110	0.38241	125.337	140.631	7.30	18.20
	C	128	132.094	254.120	0.22461	127.649	136.538	3.60	18.40
	D	466	121.281	288.953	0.13386	118.651	123.912	3.60	19.60
	Total	674	124.534	285.948	0.11014	122.372	126.697	3.60	19.60
**Platelets (*p* = 0.083)**	A	18	290,222.22	137.562.630	32.423.823	221,813.94	358,630.51	120,000	616,000
	B	62	261,435.48	128.259.294	16.288.947	228,863.72	294,007.25	88,000	696,000
	C	128	294,492.19	114.611.634	10.130.333	274,446.09	314,538.29	41,000	771,000
	D	466	261,748.93	138.681.484	6.424.296	249,124.68	274,373.17	5000	893,000
	Total	674	268,698.81	133.841.626	5.155.387	258,576.24	278,821.39	5000	893,000
**Prothrombin time (PT) (*p* = 0.542)**	A	14	132.571	197.317	0.52735	121.179	143.964	10.10	17.10
	B	29	140.955	388.064	0.72062	126.194	155.716	10.00	28.50
	C	63	131.302	482.225	0.60755	119.157	143.446	10.10	47.90
	D	228	151.105	1.205.438	0.79832	135.375	166.836	9.90	158.50
	Total	334	145.712	1.027.064	0.56198	134.657	156.767	9.90	158.50
**Thromboplastin time (TPT) (*p* = 0.004)**	A	14	28.207	49.644	13.268	25.341	31.074	15.1	36.0
	B	28	31.954	74.093	14.002	29.081	34.827	24.1	55.0
	C	62	30.606	43.014	0.5463	29.514	31.699	11.3	41.4
	D	224	32.921	63.902	0.4270	32.080	33.763	17.1	61.3
	Total	328	32.200	61.917	0.3419	31.527	32.873	11.3	61.3
**Creatinine (*p* = 0.287)**	A	18	17.311	153.138	0.36095	0.9696	24.927	0.50	6.32
	B	61	14.628	174.901	0.22394	10.148	19.107	0.32	9.51
	C	124	14.533	227.660	0.20444	10.486	18.580	0.34	20.20
	D	458	18.507	242.857	0.11348	16.277	20.737	0.28	27.00
	Total	661	17.371	232.795	0.09055	15.593	19.149	0.28	27.00
**P/F ratio (*p* = 0.018)**	A	15	280.60	96.573	24.935	227.12	334.08	70	448
	B	47	284.09	60.568	8.835	266.30	301.87	132	429
	C	77	317.94	57.594	6.563	304.86	331.01	85	448
	D	349	294.67	70.118	3.753	287.29	302.05	48	583
	Total	488	296.89	68.870	3.118	290.76	303.01	48	583
**Bilirrubin (*p* = 0.616)**	A	9	12.567	142.815	0.47605	0.1589	23.544	0.26	4.80
	B	45	0.9471	107.171	0.15976	0.6251	12.691	0.15	4.93
	C	77	10.195	181.664	0.20703	0.6072	14.318	0.14	10.76
	D	303	12.858	223.682	0.12850	10.330	15.387	0.10	19.70
	Total	434	12.029	205.935	0.09885	10.086	13.971	0.10	19.70

**Table 4:** Prediction of mortality among subphenotypes.

	B	Standard Error	Sig.	OR	95% C.I. for OR	
					Inferior	Superior
Subphenotypes			0.038			
A	2.120	0.861	0.014	8.333	1.541	45.052
B	1.056	0.780	0.176	2.874	0.623	13.257
D	1.563	0.604	0.010	4.773	1.462	15.587
C ^1^				1.000		
(Constant)	−3.730	0.584	0.000	0.024		

^1^ Reference group.

## Data Availability

The data supporting the findings of this study will be deposited in the Fundación Universitaria Sanitas Institutional Repository and will be made publicly available upon acceptance of the manuscript. The repository platform is available at https://repositorio.unisanitas.edu.co/home (accessed on 10 November 2025).

## Supplementary Materials

The following supporting information can be downloaded at: https://www.mdpi.com/article/10.3390/jcm15062264/s1, Table S1: Variables Table.

## Abbreviations

The following abbreviations are used in this manuscript:

CRP	C-Reactive Protein
GBTM	Group-based trajectory model
ANOVA	Analysis of variance
