# Supplementary material for: Predictive Model of Dynamic Subphenotypes for 30-Day Mortality in Emergency Department Patients with Suspected Infection Using the Vital Signs of the First 24 Hours: An Analytical Cohort Study in a Tertiary Care Clinic
Source: J Clin Med. 2026 Mar 17;15(6):2264. doi: 10.3390/jcm15062264 (PMC13026691; doi:10.3390/jcm15062264)
Supplement: Supplementary file 1 [file jcm-15-02264-s001.zip › jcm-3822526-supplementary.pdf]

Table S1 VARIABLES TABLE

Variable	Operational Definition	Measurement Unit	Type	Measurement Level/Scale	Variable Characterization
Age	Completed years of age at the beginning of follow-up	Years	Quantitative discrete	Numerical	Confounding
Sex	Biological characteristics of the individual	0. Female / 1. Male	Qualitative nominal	Dichotomous	Confounding
Heart rate	Number of heartbeats per minute (F1, F2, F3, F4); 4 measurements in 24 hours	Beats per minute measured by pulse oximeter or manually by nurse/physician in triage	Quantitative discrete	Numerical	Confounding
Respiratory rate	Number of breaths per minute (R1, R2, R3, R4); 4 measurements in 24 hours	Breaths per minute counted by nurse/physician	Quantitative discrete	Numerical	Confounding
Blood pressure	Force exerted by blood	mmHg by auscultatory method	Quantitative discrete	Numerical	Confounding

	against arterial walls (P1–P4); 4 measurements in 24 hours	(Korotkoff sounds) or monitor reading			
Body temperature	Body temperature in Celsius (T1–T4); 4 measurements in 24 hours	°C measured with thermometer	Quantitative continuous	Numerical	Confounding
Renal replacement therapy	Extracorporeal blood detoxification within first 96h of admission	0. Yes / 1. No	Qualitative nominal	Dichotomous	Confounding
Mechanical ventilation	Use of ventilator support within 48h of admission	0. Yes / 1. No	Qualitative nominal	Dichotomous	Confounding
Vasopressor use	Vasopressor administered within first 48h of admission	0. Yes / 1. No	Qualitative nominal	Dichotomous	Confounding
Inotrope use	Inotrope administered within first 48h of admission	0. Yes / 1. No	Qualitative nominal	Dichotomous	Confounding

Heart failure	Clinical diagnosis of structural/functional cardiac abnormality	0. Yes / 1. No	Qualitative nominal	Dichotomous	Confounding
COPD	Chronic obstructive pulmonary disease documented in chart	0. Yes / 1. No	Qualitative nominal	Dichotomous	Confounding
Hypertension	Blood pressure >140/90 mmHg	0. Yes / 1. No	Qualitative nominal	Dichotomous	Confounding
Chronic kidney disease	Structural/functional renal alteration >3 months	0. Yes / 1. No	Qualitative nominal	Dichotomous	Confounding
Liver disease	Presence of one or more liver function abnormalities	0. Yes / 1. No	Qualitative nominal	Dichotomous	Confounding
Pneumonia	Infection/inflammation of lung parenchyma	0. Yes / 1. No	Qualitative nominal	Dichotomous	Confounding

Intra-abdominal infection	Infection within abdominal cavity	0. Yes / 1. No	Qualitative nominal	Dichotomous	Confounding
Urinary tract infection	Microorganisms in urinary tract with symptoms	0. Yes / 1. No	Qualitative nominal	Dichotomous	Confounding
Skin and soft tissue infection	Inflammatory signs in skin/subcutaneous tissue	0. Yes / 1. No	Qualitative nominal	Dichotomous	Confounding
CNS infection	Infection involving the central nervous system	0. Yes / 1. No	Qualitative nominal	Dichotomous	Confounding
Infective endocarditis	Infection of endocardial tissue (valves or cavities)	0. Yes / 1. No	Qualitative nominal	Dichotomous	Confounding
Catheter-related infection	Same microorganism in ≥3 blood cultures including catheter tip	0. Yes / 1. No	Qualitative nominal	Dichotomous	Confounding

Infection of unknown origin	Signs/symptoms of infection with no defined focus	0. Yes / 1. No	Qualitative nominal	Dichotomous	Confounding
Acute myocardial infarction	Myocardial damage with ischemia and troponin rise above 99th percentile + criteria (ECG, imaging, etc.)	0. Yes / 1. No	Qualitative nominal	Dichotomous	Confounding
Peripheral vascular disease	Stenosis of peripheral arteries/veins	0. Yes / 1. No	Qualitative nominal	Dichotomous	Confounding
Cerebrovascular disease	Focal brain dysfunction due to imbalance between O_2 supply and demand	0. Yes / 1. No	Qualitative nominal	Dichotomous	Confounding
Connective tissue disease	Chronic inflammatory/autoimmune disease affecting multiple organs	0. Yes / 1. No	Qualitative nominal	Dichotomous	Confounding

Peptic ulcer	Ulcer in stomach or duodenal mucosa	0. Yes / 1. No	Qualitative nominal	Dichotomous	Confounding
Diabetes mellitus	HbA1c ≥6.4% or random glucose >200mg/dL or fasting glucose ≥126mg/dL or 2h OGTT >200mg/dL	0. Yes / 1. No	Qualitative nominal	Dichotomous	Confounding
Leukemia	Malignant hematologic disorder affecting blood cells	0. Yes / 1. No	Qualitative nominal	Dichotomous	Confounding
Lymphoma	Malignant tumor of lymphatic tissue	0. Yes / 1. No	Qualitative nominal	Dichotomous	Confounding
Solid metastasis	Secondary neoplastic mass in other anatomical region	0. Yes / 1. No	Qualitative nominal	Dichotomous	Confounding

AIDS (HIV disease)	Chronic stage of HIV infection	0. Yes / 1. No	Qualitative nominal	Dichotomous	Confounding
Leukocytes	White blood cell count in peripheral blood	Cells/µL	Quantitative continuous	Numerical	Confounding
Absolute neutrophil count	Neutrophil count in peripheral blood	Cells/µL	Quantitative continuous	Numerical	Confounding
Lymphocytes	Lymphocyte count in peripheral blood	Cells/µL	Quantitative continuous	Numerical	Confounding
Neutrophil-to-lymphocyte ratio (NLR)	Ratio of neutrophil to lymphocyte counts — systemic inflammation predictor	Cells/µL	Quantitative continuous	Numerical	Confounding
C-reactive protein (CRP)	Plasma protein produced by liver in inflammation	mg/dL	Quantitative discrete	Numerical	Confounding
Erythrocyte sedimentation rate (ESR)	Rate of RBC sedimentation in 1 hour	mm/sec	Quantitative continuous	Numerical	Confounding

Lactic acid	Product of anaerobic glucose metabolism	mmol/L	Quantitative discrete	Numerical	Confounding
Hemoglobin	Oxygen-transporting protein in red blood cells	g/dL	Quantitative discrete	Numerical	Confounding
Platelets	Cells responsible for hemostasis	Cells/μL	Quantitative continuous	Numerical	Confounding
Prothrombin time	Coagulation test of extrinsic pathway	Seconds	Quantitative discrete	Numerical	Confounding
Partial thromboplastin time	Coagulation test of intrinsic pathway	Seconds	Quantitative discrete	Numerical	Confounding
Creatinine	Final product of creatine metabolism	mg/dL	Quantitative discrete	Numerical	Confounding
PaO_2/FiO_2 ratio (PAFI index)	Ratio between arterial O_2 pressure and inspired O_2 fraction	mmHg	Quantitative continuous	Numerical	Confounding

Bilirubin	Waste product from red blood cell breakdown	mg/dL	Quantitative discrete	Numerical	Confounding
30-day mortality	Death from any cause within 30 days after inclusion	0. Yes / 1. No	Qualitative nominal	Dichotomous	Dependent
Vital signs trajectory phenotypes	Groups of patients with similar vital-sign trajectories and suspected infection	Groups A–D (based on HR, RR, BP, Temp)	Quantitative continuous	Numerical	Independent
